# A multi-biomarker micronucleus assay using imaging flow cytometry

**DOI:** 10.1007/s00204-024-03801-7

**Published:** 2024-07-12

**Authors:** Danielle S. G. Harte, Anthony M. Lynch, Jatin Verma, Paul Rees, Andrew Filby, John W. Wills, George E. Johnson

**Affiliations:** 1 Swansea University Medical School, Swansea University, Swansea, UK; 2 GSK R&D, Stevenage, UK; 3 College of Engineering, Swansea University, Swansea, UK; 4 Imaging Platform, Broad Institute of MIT and Harvard, Cambridge, MA USA; 5 Core Flow Facility, Faculty of Medical Sciences, Newcastle University, Newcastle Upon Tyne, UK; 6 Department of Veterinary Medicine, Cambridge University, Cambridge, UK

**Keywords:** ImageStream, Micronucleus, NAM, DNA damage, MoA, Biomarker

## Abstract

Genetic toxicity testing assesses the potential of compounds to cause DNA damage. There are many genetic toxicology screening assays designed to assess the DNA damaging potential of chemicals in early drug development aiding the identification of promising drugs that have low-risk potential for causing genetic damage contributing to cancer risk in humans. Despite this, in vitro tests generate a high number of misleading positives, the consequences of which can lead to unnecessary animal testing and/or the abandonment of promising drug candidates. Understanding chemical Mode of Action (MoA) is vital to identifying the true genotoxic potential of substances and, therefore, the risk translation into the clinic. Here we demonstrate a simple, robust protocol for staining fixed, human-lymphoblast p53 proficient TK6 cells with antibodies against ɣH2AX, p53 and pH3S28 along with DRAQ5™ DNA staining that enables analysis of un-lysed cells via microscopy approaches such as imaging flow cytometry. Here, we used the Cytek® Amnis® ImageStream®X Mk II which provides a high-throughput acquisition platform with the sensitivity of flow cytometry and spatial morphological information associated with microscopy. Using the ImageStream manufacturer’s software (IDEAS® 6.2), a masking strategy was developed to automatically detect and quantify micronucleus events (MN) and characterise biomarker populations. The gating strategy developed enables the generation of a template capable of automatically batch processing data files quantifying cell-cycle, MN, ɣH2AX, p53 and pH3 populations simultaneously. In this way, we demonstrate how a multiplex system enables DNA damage assessment alongside MN identification using un-lysed cells on the imaging flow cytometry platform. As a proof-of-concept, we use the tool chemicals carbendazim and methyl methanesulphonate (MMS) to demonstrate the assay’s ability to correctly identify clastogenic or aneugenic MoAs using the biomarker profiles established.

**Supplementary Information:**

The online version contains supplementary material available at 10.1007/s00204-024-03801-7.

## Introduction

Genotoxicity is the property of a chemical or physical agent to cause DNA or chromosomal damage. The discipline of genetic toxicology is responsible for the assessment of compounds or agents, and/or their respective metabolites, to damage genetic information either directly or indirectly. The Organisation for Economic Co-operation and Development (OECD) provides guidance for regulatory genetic toxicology testing in order to unify assay standards for all member countries worldwide. One of the mandated assays in genetic toxicology test battery is the “In vitro mammalian cell micronucleus (MN) test” (OECD Test No. 487) which was developed for the detection of micronuclei in the cytoplasm of interphase cells and provides a measure of chromosomal DNA damage—a recognised key event in the initiation of cancer (Fenech 2000, 2020; Fenech et al. 2003; OECD 2023).

Micronuclei may originate from acentric chromosome fragments (i.e., those lacking a centromere), or whole chromosomes that are unable to migrate to the mitotic poles during the anaphase stage of cell division. Therefore, the in vitro micronucleus (MNvit) test is an in vitro method that provides a comprehensive basis for investigating chromosome damaging potential in vitro because both aneugens and clastogens can be detected (Countryman and Heddle 1976; Fenech 2007; Kirsch-Volders 1997; Schmid 1975). Typically, micronucleated cells have been assessed using microscopy, via either manual scoring or semi-automated image-analysis classifiers, but more recently, flow cytometry approaches have been widely utilised (Avlasevich et al. 2011). Whereas manual scoring can be highly laborious, there are concerns that automated methods can potentially lead to misleading positive or negative outputs, through over- or under-scoring respectively (Johnson et al. 2014; Verma et al. 2017). One of the main challenges with the MNvit test, in its various incarnations, is the reported high frequency of misleading positives, especially when using certain mammalian cell types (Fowler et al. 2012). In this context, misleading positives are those chemicals that are not confirmed as positive in subsequent rodent in vivo micronucleus tests. As a consequence, unnecessary animal studies may be conducted and/or the development of promising compounds abandoned. In these circumstances, further in vitro testing may be required to characterise the Mode of Action (MoA) responsible for MN formation in an attempt to clarify translational relevance for human risk assessment.

**Fig. 1:**
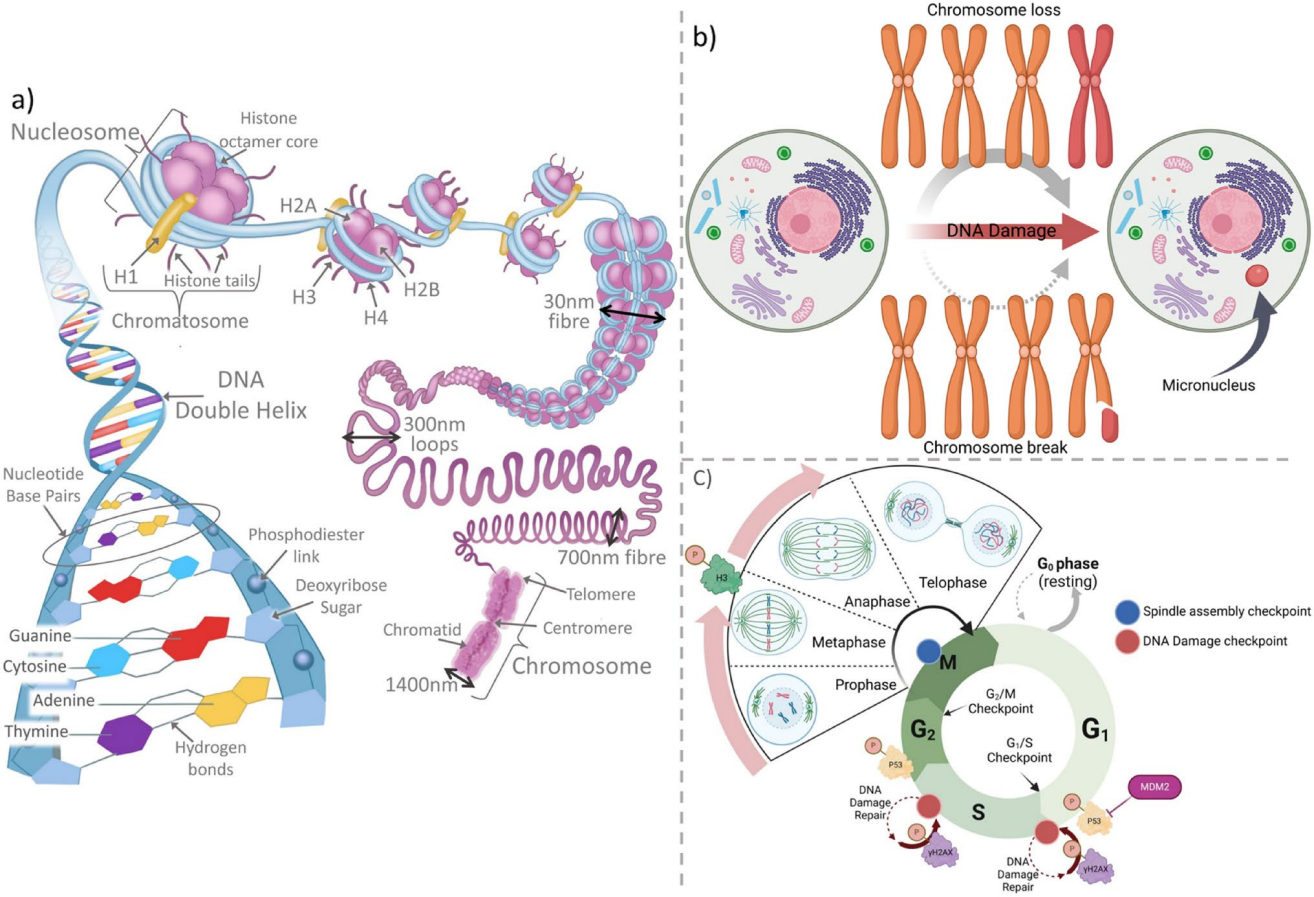
DNA structure, micronucleus formation and cell-cycle biomarker relationships. **A** Schematic showing the primary, secondary and tertiary structure of DNA. DNA wraps around histone octamer protein cores consisting of H2A, H2B, H3 and H4 dimers, forming the nucleosome. Nucleosome folding compacts DNA to form the chromatid arm, yielding a metapahse chromosome formed of two sister chromatids joined at the centromere. **B** Routes of micronucleus induction. Compound DNA interaction can lead to MN formation by either aneugenic (chromosome loss) or clastogenic (chromosome breakage) routes. Chromosome break or loss results in a MN as the DNA segments are not pulled to the poles and thus remain in the cytoplasm when the nuclear envelope reforms and cytokinesis occurs. The red coloured chromosome/chromosome piece indicates the DNA content that becomes the MN (also shown in red). **C** Cell cycle and biomarker activation. Upon DNA damage, p53 is phosphorylated resulting activation of DNA damage repair pathways and cell cycle arrest. Double-strand and single-strand breaks lead to the formation of DNA repair complexes signalled by epigenetic modifications such as H2AX phosphorylation. Successful repair of DNA damage may allow the cell cycle to continue. Phosphorylation of H3 signals chromatin re-organisation including prophase initiation, global phosphorylation at metaphase and progression into anaphase (**B**, **C** Created with BioRender.com)

Conventionally, the presence or the absence of centromeric labels within the MN have been used to discriminate between aneugenic or clastogenic MoAs and inform risk assessment (OECD 2023). Recently, additional molecular biomarkers have been used to identify MoA, for example, the increase in phosphorylated histone 3 (pH3) is associated with increased aneuploidy, whilst phosphorylated histone variant H2AX (ɣH2AX) is an indicator of clastogenicity (Audebert et al. 2010; Bryce et al. 2016; Cheung et al. 2015). Phosphorylation of H2AX, on serine 139 of the SQEY tail, upon strand breakages results in the activation of DNA damage repair, with the ɣH2AX foci occurring in a 1:1 ratio with regard to DNA damage (Hoeller and Dikic 2009; Watters et al. 2009; Zhou et al. 2006). To ensure movement through mitosis, chromosome condensation is accompanied by phosphorylation of the H3 protein, at two different serine residues, S10 and S28 (Doerig et al. 2015; Hans and Dimitrov 2001). Phosphorylation of H3 at the S28 position begins at prophase and by late anaphase S28 is completely dephosphorylated (Hans and Dimitrov 2001). As such, the characteristics of pH3 and ɣH2AX, used in combination with immunofluorescent antibodies (AB) alongside the MNvit assay, has been applied to flow-cytometry platforms to determine the clastogenic/aneugenic potential of compounds (Bryce et al. 2016; Smart et al. 2011). Other biomarkers, e.g., the tumour suppressor protein p53 have been used to assess DNA damage cell response. P53 complements the specific biomarkers of aneugenicity and clastogenisity given its intrinsic role in the DNA damage response and cell cycle progression (Bryce et al. 2016; Lavin and Gueven 2006) (Fig. 1).

The recent development of imaging flow cytometry platforms combines the high-throughput data acquisition with the spatial image morphology information and archiving capabilities of automated microscopy (Allemang et al. 2021; Rodrigues 2018, 2019; Rodrigues et al. 2014, 2016a, b, 2018; Wang et al. 2019; Wilkins et al. 2017). Cell imagery provides increased confidence in flow-cytometry gating strategies and the development of improved imaging classifiers and scoring precision, since captured images can be used to refine and validate gating approaches.

MN enumeration using ImageStream flow cytometry has been reported for the binucleated cytokinesis-block micronucleus (CBMN) assay using fluorescent DNA staining (e.g., DAPI, Hoechst and DRAQ5™) (Rodrigues et al. 2016a; Verma et al. 2018; Wills et al. 2021). However, MN assessment using the mononuclear MN assay incorporating additional mechanistic biomarkers (e.g., ɣH2AX, pH3, p53) has not been reported using the ImageStream platform. To enable this here, immunocytochemical staining methods were optimised enabling assessment of un-lysed cells by ImageStream cytometry with a subsequent, automated scoring approach developed using the ‘IDEAS®’ software. In this way, this paper reports an automated, in vitro micronucleus assay incorporating automated scoring of ɣH2AX, pH3 and p53 DNA damage events plus cell-cycle information using the ImageStream platform (ISMN-mb assay). Template-based, batch processing is demonstrated for high-throughput automation, dose–response generation and MoA classification using the tool compounds methane methyl sulphonate (MMS) and carbendazim. The approach is compared to mechanistic assays run using traditional flow cytometry and the current limitations and future potential of the method is discussed.

## Results

The ImageStream flow cytometer enables rapid collection of single cell images (~ 7000 per minute) from multiple fluorescence channels (6 +). Following treatment with a tool clastogen (MMS) and/or aneugen (carbendazim), cells were harvested, fixed and stained prior to analysis. An image capture process for the acquisition of single cell images for cell samples with concurrent DNA staining and ɣH2AX, p53, and pH3 biomarker labelling was developed along with an image analysis pipeline using the cytometer manufacturer’s software (IDEAS®) that enables micronucleus detection, cell cycle assessment alongside simultaneous quantification of the three DNA damage biomarkers. The template-based gating strategy for automated biomarker and cell cycle assessment are hereby described in detail.

### Micronucleus detection

**Fig. 2:**
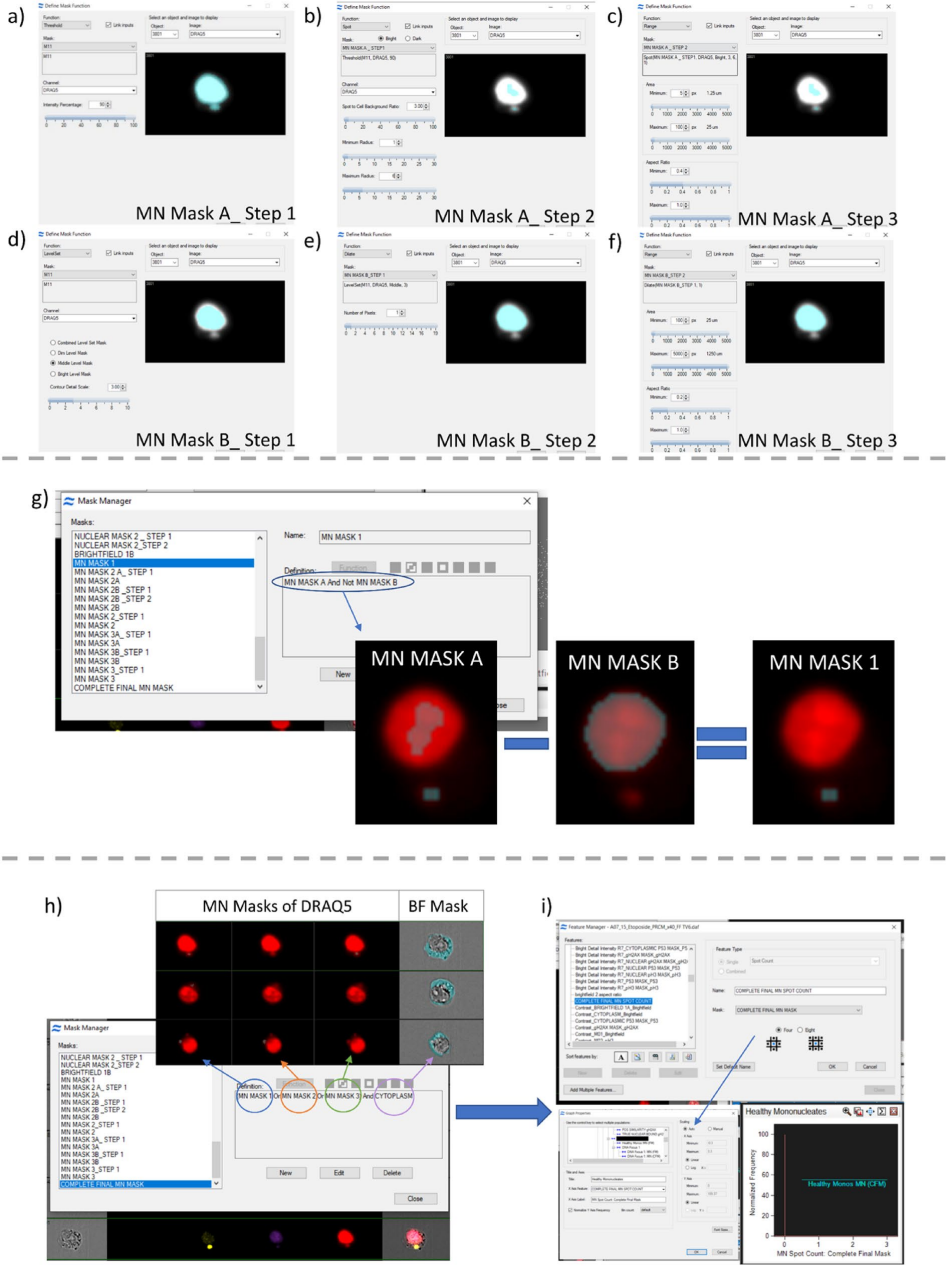
Nucleus and Micronucleus segmentation strategy in the IDEAS software. **a**–**f** Step-wise approach for generating the masks, ‘MN Mask A’ and ‘MN Mask B’, that combine to make ‘MN Mask 1’. **a** Intensity thresholding on the DNA stain channel removed 10% of the lower intensity pixels to generate ‘MN Mask A_Step 1’. **b**/**c** Shows how the MN events (MN Mask A) in MN Mask 1 were segmented. **b** The ‘Spot Mask’ function was applied to the pixels within MN MASK A_Step 1. Setting a low spot to background ratio extracted the brightest spots in the image regardless of the intensity differences between them. The minimum and maximum radius settings identified bright spots between 1–6 pixels in diameter (MN MASK A_Step 2). **c** The Range function was then applied to MN MASK A_Step 2. Setting the area minimum and maximum applied a size limitation to the spots identified, set at 1.25–25 µm. The aspect ratio setting defined criteria for the “roundness” of spots being segmented and was set at 0.4–1. **d**–**f** Demonstrates how the parent nucleus was segmented (MN Mask part B). **d** Using the ‘Level Set’ function at medium brightness level and contour detail level of 3 set the mask tight to the nuclear morphology (MN MASK B_Step1). **e** The dilate function was then applied to extend the mask boundary by 1 pixel (MN MASK B_ Step2) to better capture the outer boundary of the nucleus. **f** The Range function was then used to limit the size and shape range of identified nuclei. The minimum area value was set to the maximum area value in MN Mask A i.e. objects larger than any MN event were masked as parent nuclei. **g** Shows how MN MASK A and MN MASK B were subtracted using Boolean logic to yield MN MASK 1. This process was repeated for each of the three MN masks. **h** Combination of the three MN masks yielding the ‘Complete Final MN’ mask used for micronucleus segmentation. Demonstrates the segmentation achieved for each of the three MN masks. Use of the OR command enables all three masks to be used in combination. Use of the AND command restricts MN instances to those within the cytoplasm region. **i** Demonstrates use of ‘Feature Manager’ to plot a histogram of the number of MN events per cell

Cell nuclei and MN were detected using nuclear-stained images measured using the imaging flow cytometer. Using the IDEAS® 6.2 software a series of defined image masks were combined in a stepwise manner (Fig. 2a–f). An intensity threshold was first set on the nuclear channel to identify the DRAQ5 nuclear-stained regions of the image (i.e., parent nuclei and MN events). The ‘Spot’ mask function was used to isolate all bright spots in the image. These spots were filtered using roundness and size criteria to identify potential MN events (Fig. 2a–c). To exclude spots originating inside the parent nucleus, the isolation of the main nucleus was required. Therefore, ‘Level Set’ and ‘Range’ functions were used to identify larger, main parent-nuclei (Fig. 2d–f). Subtraction of the parent nucleus mask ensured that spots filtered as MN events would lay outside of the parent nucleus (Fig. 2g). This MN masking process was repeated three times using different size and roundness settings to maximise the sensitivity of MN detection. It was found that increasing the complexity of the mask past 3 iterations, ‘MN MASK 1, 2 and 3’, offered no additional benefit to the extraction of MN events. The MN masks 1, 2 and 3 were then combined with the ‘cytoplasm mask’ (determined as everything below the nuclear intensity threshold) to generate the ‘Complete Final MN Mask’ (CFM) (Fig. 2h).

To automate MN detection, the IDEAS® ‘Spot Count’ function was deployed within the CFM mask on the healthy, mononucleated cell population (defined as acceptably focussed, single, round, DNA-stained cells with no pH3 staining). This generated a histogram organising the cell population according to frequency of MN events (Fig. 2i). To assess accuracy (i.e., correctly identified MN events) and miss rate (i.e., MN events not identified) by the automated analysis process, approximately four thousand cells originating from four different raw image files were manually assessed. Comparison of the results showed the mean accuracy for automated MN detection was 57% (range 35–72%) with a miss rate of 44% (range 3–73%). Whilst discussed in greater detail below, this benchmarking against manual scoring highlights the limitations of a simple, threshold-based masking system.

### Cell cycle and biomarker gating

**Fig. 3:**
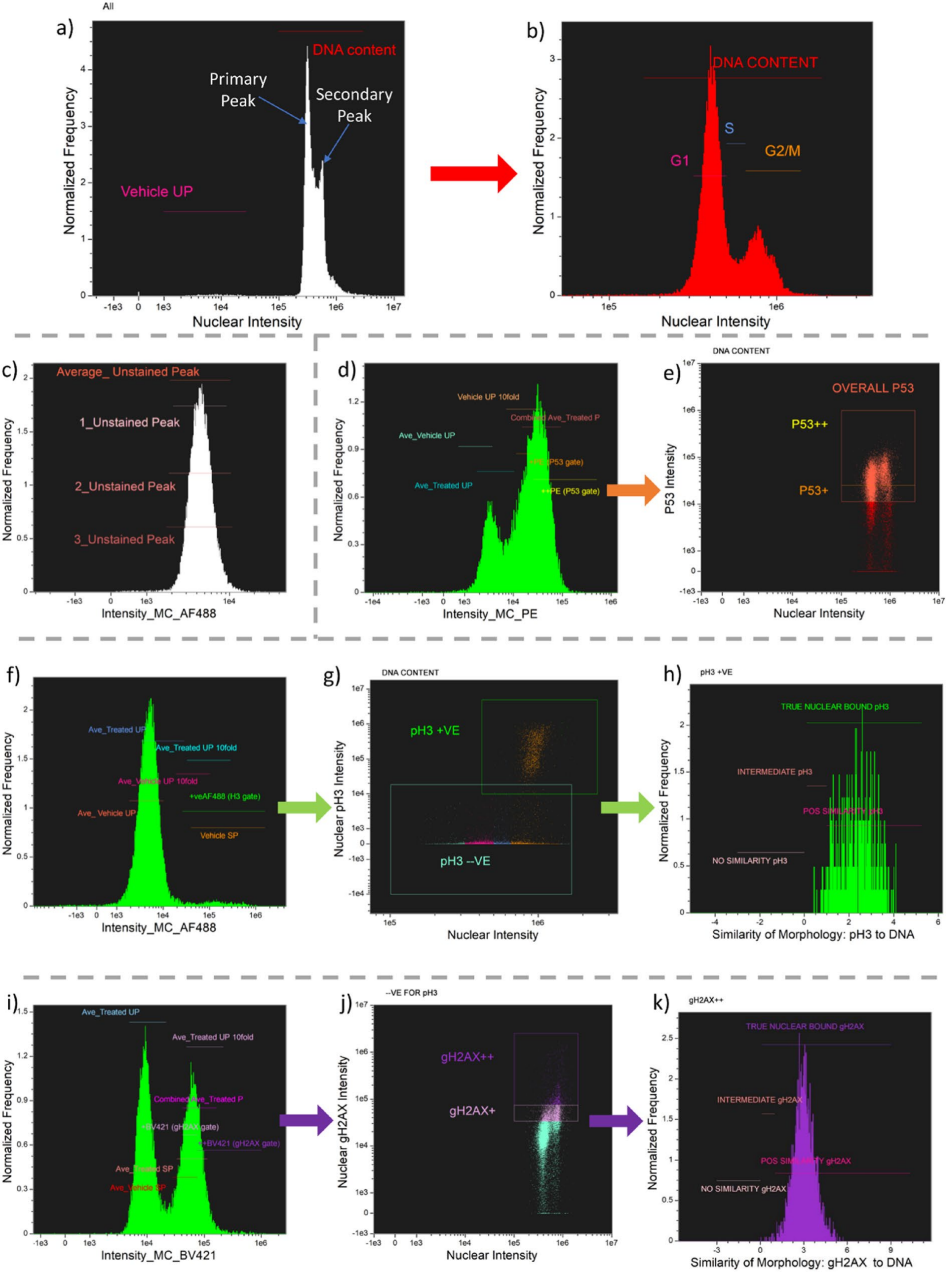
Gating strategy for cell cycle, p53, pH3 and ɣH2AX assessment. **a** Intensity histogram for DNA-stained cells showing gates for the ‘unstained population’ (UP) and events with DNA staining. **b** Gates were created for G1, S and G2/M cell cycle positions. The G1 gate (nuclear content *n* = 1) was positioned over the ‘primary peak’. The G2 gate was set by shifting the G1 gate upward by ~ 1.8–2×. Guided by the pH3 mitotic marker, an M gate was created. Its lower boundary was then combined with the G2 gate, providing the final G2/M population. The S gate was then defined as the region between the G1 and G2/M gates. **c** Fluorescence histogram for an unstained cell sample enabling positioning of an unstained peak gate. To define a gate for capturing positive PE (P53), AF488 (pH3) and BV421 (gH2AX) events (shown, **d**, **f**, **i**), this unstained peak gate was shifted to the right by a factor of 10. **d**, **e** Gating strategy for p53 positive events. **d** The ‘ Vehicle UP tenfold’ gate lower boundary was set 10% lower than the maximum autofluorescence determined from the US treated peak. **e** Final gate (‘overall P53’) used for p53 assessment. **f**–**h** Gating strategy for pH3 gate generation. **f** The ‘Vehicle SP’ gate was set over the stained secondary peak (shown **f**). due to the definitive nature of the PH3 biomarker, this gate was then combined with the ‘Average tenfold gate’ lower boundary to give the ‘ + veAF488 (H3 gate)’. **g** Final gate position defining the ‘pH3 + VE’ population. Underneath, the ‘pH3 −VE’ population is shown. This population is highlighted according to cell cycle position (gates shown in **b**). The pH3 + ve population is distinctly separated and is seen to sit over the G2/M population as expected. **h** Cytoplasmic and nuclear pH3 signal was separated using the ‘similarity of morphology’ feature (exemplified, Fig. 4). Increasing signal overlap with the nuclear mask increases the similarity of morphology score. In this way the ‘True Nuclear bound pH3’ population was gated excluding any cytoplasmic or off target signal. **i**–**k** Gating strategy for ɣH2AX populations. **i** Relative to cells not expressing gH2AX (primary peak) the + BV421 (gH2AX) and +  + BV421 (gH2AX) were positioned on the secondary peak. The positioning of these gates was informed by samples exposed or unexposed to a clastogen to separate background gH2AX expression from clastogenically-induced DNA damage. **j** Final gates used for gH2AX assessment. **k** The gH2AX +  + gated population was carried forward and refined to reflect the nuclear located gH2AX events yielding the ‘True Nuclear bound gH2AX’ population using similarity of morphology feature. Example raw data and a template file enabling reproduction of the gating strategy is provided for download at the BioStudies database under accession number S-BSST1351

The next step was the classification of the cell images captured into populations representing the different cell-cycle stages alongside quantification of the immunofluorescence data for the pH3, ɣH2AX and p53 biomarkers. To determine the cell-cycle positions, the intensity of the DRAQ5 nuclear fluorescence was first used to gate the DRAQ5-positive cell population from the unstained background/debris events (Fig. 3a, ‘DNA content’ gate). A histogram showing the nuclear fluorescence per cell for this population then revealed the characteristic G1, S and G2/M cell-cycle positions enabling cell-cycle gate placements (Fig. 3b).

Biomarker quantification (ɣH2AX, pH3 and p53) followed the same basic methodology for each immunofluorescence marker. The first step used unstained samples to determine background autofluorescence levels by plotting an intensity histogram for each biomarker channel for the single, in-focus cell population. Using data from multiple unstained samples with and without genotoxicant treatment, gates were repeatedly positioned to capture the ‘unstained peak’ (UP) and the average position determined (Fig. 3c). The coordinates of this unstained peak gate were then shifted upwards by a factor of 10 to define a gated region (termed the ‘UP tenfold’ gate) of expected fluorescence for stained samples (Fig. 3d, f, i). The peaks present in stained samples were then used to refine these gate positions further, yielding the ‘stained sample peak’ (SP) gates. In each instance, the coordinates of these SP gates were averaged with the coordinates of the UP tenfold gates to provide the final optimised, ‘Combined Average’ gate capturing each stained cell population (Fig. 3 d, f, i).

### p53 Gating (phycoerythrin label)

Expression of p53 was measured using a phycoerythrin (PE) conjugated antibody that is specific to the N-terminal region of the protein thus maximising the detectability of different isoforms. To quantify the p53-positive cell population, the difference between the ‘Vehicle UP tenfold’ and ‘Combined Ave_Treated P’ gates (described above) on the PE channel was used to generate a ‘ + PE (P53 gate)’ and ‘ +  + PE (P53 gate)’ gate (Fig. 3d). These gates were then plotted against the nuclear intensity of each cell (Fig. 3e) allowing gating of the final ‘Overall P53’ population to encompass all events positive for p53 that also exhibited DNA staining (Fig. 3e). Given that constitutive expression of p53 was detectable in unexposed cells, separation of p53-positive events into ‘P53 + ’ (background p53 levels) and ‘P53 +  + ’ (induced cell populations) was considered potentially useful for downstream analyses (e.g., relative expression under different exposures with cell cycle position, etc.). In the present work, however, the ‘OVERALL P53’ population is the metric used in the dose–response relationships subsequently presented in Fig. 5.

### Phosphorylated histone H3 gating (AlexaFluor 488 label)

The expression of the phosphorylated histone H3 (pH3) was measured using a phospho-specific antibody that binds the serine 28 residue of the phosphorylated protein. In contrast to the constitutively expressed p53 and YH2AX biomarkers, phosphorylation of histone H3 only occurs in late G2 and mitosis phases of the cell cycle, simplifying the gating process of pH3-positive events.

**Fig. 4:**
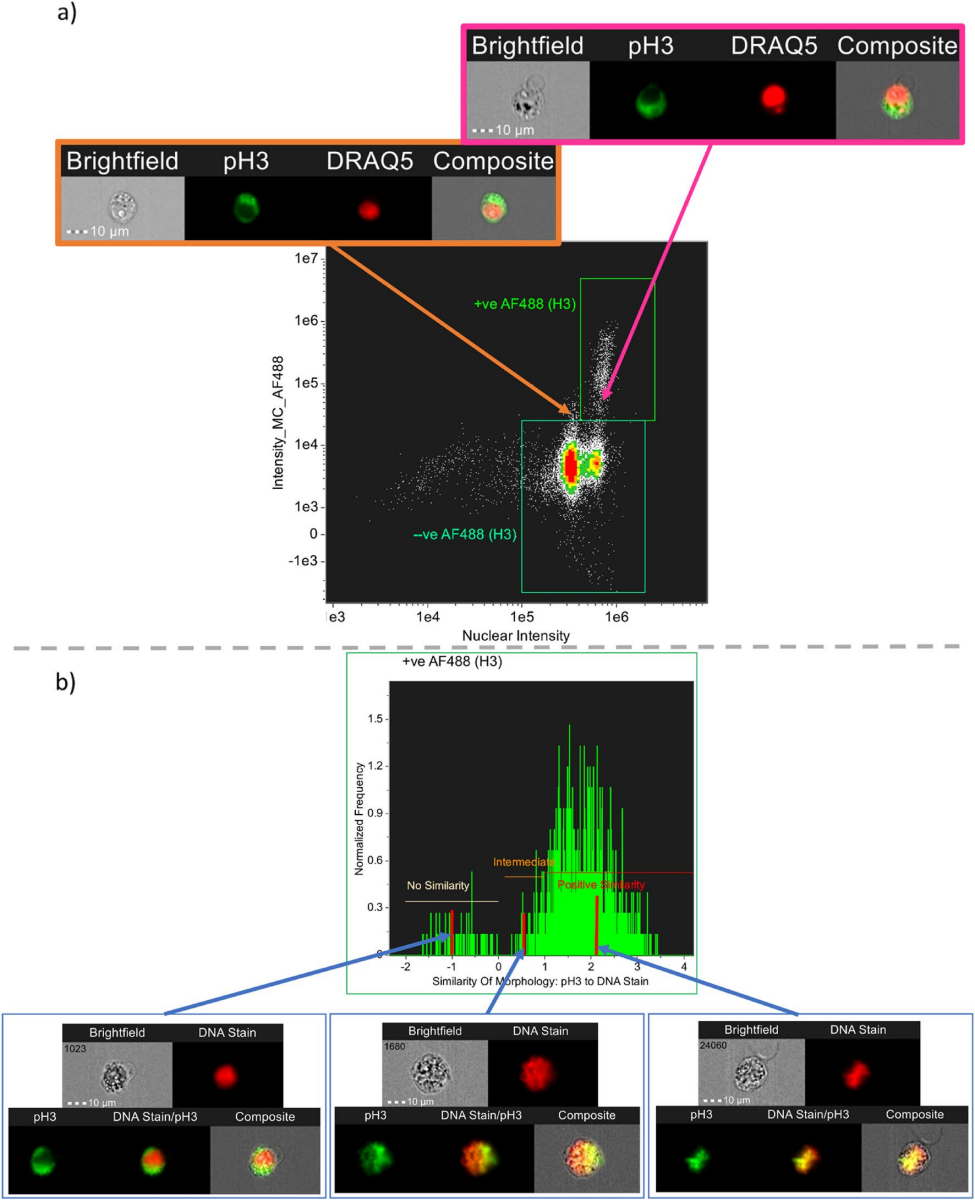
Use of similarity of morphology to refine nuclear and cytoplasmic signals. **A** Scatter graph for a carbendazim sample (1.6 µg/mL) showing pH3 gating. The orange inset shows exclusion of events with signal outside the nucleus. However, the pink inset shows that events with cytoplasmic staining are still present. **B** Similarity of morphology was used to describe the degree of overlap between the signal and nuclear mask. The insets demonstrates events with different similarity of morphology scores. Scores < 0: pH3 signal is predominantly cytoplasmic, this population was termed ‘No Similarity’. Scores 0–1: Partial signal overlap with the nucleus, population termed ‘Intermediate’. Scores > 1: Signal predominantly exhibits nuclear localisation, this population was termed ‘Positive Similarity’

Using a stained vehicle sample (Fig. 3f), the tenfold and Vehicle SP gates (described above) were combined to generate the ‘ + veAF488 (H3gate)’ gate identifying the positively-stained events. This gate was then refined further by plotting the nuclear intensity per cell against pH3 staining in the masked, nuclear region of each cell (Fig. 3g). The plot shows a distinct population of cells with high nuclear pH3 expression and extends the + veAF488 (H3gate) to encompass all cells with DNA staining and positive pH3-staining. This refined gate was termed ‘pH3 + VE’. The separation also allowed robust gating of the pH3-negative (‘pH3 −VE’) population (see Fig. 3g). This population is important to the YH2AX scoring because H2AX becomes phosphorylated in the course of normal mitosis activity and the ability to separate “mitosis-associated” and “DNA damage-associated” ɣH2AX signalling is important. Encouragingly, overlaying the cell populations associated with different stages of the cell cycle (Fig. 3b) in different colours confirmed the identified pH3 + ve cell population sits within the G2/M region of the cell cycle. To arrive at the final cell population used for automated scoring, one final analysis step was utilised to better distinguish between cells with predominantly nuclear-located AF488 signal. To do this, the ‘Similarity of Morphology’ process in the IDEAS® software was used to score the extent of each cell’s pH3 nuclear co-location. The process used is exemplified in Fig. 4, but briefly, increasing signal overlap with the nuclear mask was used to score increasingly nuclear co-located signal. In this way, the final ‘True Nuclear bound pH3’ population was gated (Fig. 3h) so as to better exclude any cytoplasmic or off-target AF488 signal providing the final population for automated scoring.

### Phosphorylated H2A histone family member X (ɣH2AX) gating (BV421 label)

The anti-ɣH2AX-Ser139 antibody biomarker is associated with the DNA damage response pathway (Podhorecka et al. 2010) and reflects DNA strand breakage (i.e., clastogenicity). Here, the Brilliant™Violet (BV)421 antibody conjugate was used.

Stained samples of MMS treated and non-treated cells resulted in a histogram of BV421 intensity with two distinct peaks (Fig. 3i). Given the distinct secondary peak available from BV421 H2AX staining for both vehicle and treated samples, gates were also positioned over the secondary peak (‘Ave_Treated SP’, ‘Ave_Vehicle SP’). The’tenfold’ and ‘stained peak’ gates were combined (as described above) and the differences between the treated and vehicle samples were used to generate the ‘ + BV421 (gH2AXgate)’ and ‘ +  + BV421 (gH2AX gate)’ gates (Fig. 3i). These gates were then plotted on a scatter graph of ɣH2AX staining in the masked nuclear region of each cell against the nuclear intensity of the cells (Fig. 3j). Each gate was extended horizontally, in excess, to encompass all cells with DNA staining and positive for H2AX staining. These gates are termed gH2AX + and gH2AX +  + . The gH2AX + lower boundary and the intermediate boundary between gH2AX + and gH2AX +  + above which H2AX signal induction is considered chemically induced DNA damage. Similarity of Morphology process in the IDEAS® software was used on gH2AX +  + population, only cells that have a similarity of > 0 were used for the ɣH2AX metric, this was termed ‘True Nuclear bound gH2AX’ (Fig. 3k).

### Population refinement

For all gates generated, the images of scatter points along the borders both outside and inside the gates were interrogated. Figure 4A highlights that whilst the extensive gating procedure does exclude false staining events (orange insert) not all of the events can be excluded (pink insert) based on gating strategy alone. Based on an understanding of pH3 and ɣH2AX biology, a feature called Similarity Of Morphology was used to identify biomarker signals that were exclusively associated with the DNA signal. This IDEAS® software feature is generally used to assess the likelihood that a signal has been translocated into the nucleus. The function assigns a + ve or −ve integer to each pixel, based on how similar pixels containing the signal of interest are in spatial location to the stained DNA pixel signal, by plotting the log-transformed Pearson correlation coefficient of the DNA stain and signal of interest (IDEAS® user manual 6.2, 2015). The software places a gate of ≥ 1 on a graph and plots the available cell population as a histogram. Cells that are ≥ 1 can be described as having a high probability that the signal of interest is in the nucleus. Any signal that is found to be < 0 are cells where the signal is most likely in the cytoplasm. In the current application, the feature was used to confirm that the biomarker signal was correctly associated with the nucleus, thus off-target binding or autofluorescence cells could be excluded from the populations being assessed. Using this feature, cells with a pH3/ɣH2AX signal associated with nuclear localisation were identified (see example insets in Fig. 4B). The intermediate population of cells between 0 and 1 were included in the final population for both ɣH2AX and pH3 metrics as the phosphorylation of these biomarkers is a dynamic event and therefore at different moments in time varying levels of phosphorylation will have occurred. The TRUE NUCLEAR BOUND populations for both pH3 and ɣH2AX used for metric extraction are shown in Fig. 3h and k.

### Results with carbendazim and MMS

**Fig. 5:**
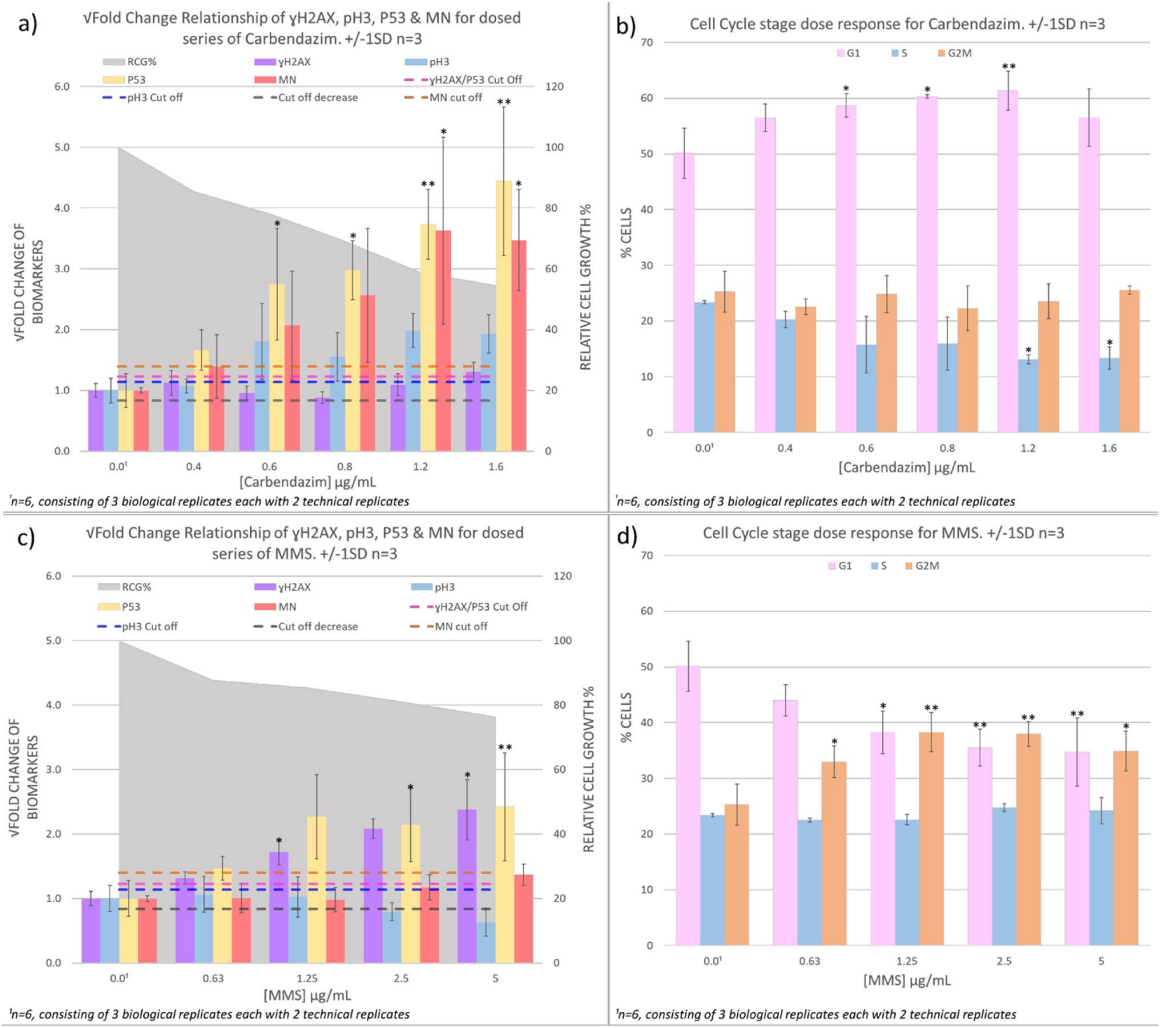
Dose–response relationships for carbendazim and methyl methane sulphonate (MMS). **a**, **c** MN, ɣH2AX, pH3 and p53 endpoints measured by imaging flow cytometry alongside relative cell growth information established from relative cell counts. Square-root transformations of the raw fold-change values were used to facilitate visualisation. Dashed lines represent the fold change cut offs which could be used to determine positive or negative calls for MN and the biomarkers pH3, p53 and ɣH2AX to inform on MoA. **b**, **d** relative proportion of cells at each cell cycle stage measured by imaging flow cytometry from nuclear intensity information. Asterisks indicate the statistical significance of responses relative to vehicle control levels (*p < 0.05 or **p < 0.005, Dunnett’s *t*-test method)

Dose–response relationships for MN induction and ɣH2AX, p53 and pH3 biomarker expression in human TK6 cells are shown following treatment with carbendazim (Fig. 5a, b) or MMS (Fig. 5c, d). Results are expressed as fold-increases compared to that the vehicle control, DMSO. In addition, % relative cell growth (RCG) and cell cycle changes are presented for each treatment (Fig. 5). Fold change cut off values have previously been defined for biomarker responses (Ando et al. 2014; Bryce et al. 2014; Garcia-Canton et al. 2013a, b; Smart et al. 2011) but specific cut-off values for the ImageStream platform have yet to be defined. For expedience, reported fold change cut off values for ɣH2AX, p53, pH3 and MN were used to define ‘positive/negative’ and ‘Mode of Action’ outcomes in the present study (see “Materials and methods” for details).

There were concentration-dependent increases in TK6 cell cytotoxicity following carbendazim treatment with 54.5% RCG observed at 1.6 µg/mL. Compared with the vehicle control, there were clear concentration dependent increases in MN frequencies and biomarker signals (pH3 and p53) and a corresponding decrease in the ɣH2AX signal (Fig. 5a). The average MN, pH3 and p53 responses exceeded the respective fold change cut off values for a positive response at concentrations of 0.6 µg/mL and above. This biomarker profile is consistent with a DNA damage response via an aneugenic MoA. Cell cycle analysis showed concentration dependent increases in the proportion of cells in G1 and a decrease in S-phase with minimal effect on G2/M (Fig. 5b).

MMS treatment resulted in a reduction in RCG relative cell growth in TK6 cells of 76.4% at 5 µg/mL (the highest concentration tested). There were concentration dependent increases in ɣH2AX and p53 biomarker signal and a corresponding decrease in pH3 (Fig. 5c). The ɣH2AX and p53 responses exceeded the cut off values for a positive response. There was an increase in MN-frequency compared with the vehicle controls, but the increases were not statistically significant at the highest concentration tested (*p* = 0.07 @ 5 µg/mL MMS). Nevertheless, the DNA damage biomarker profile is consistent with a clastogenic MoA. Cell cycle response for MMS demonstrates a dose dependent decrease in G1 and increase G2/M cell cycle phases with minimal effect on S-phase (Fig. 5d). The absence of a MN-response probably reflected the limited concentration range of MMS tested (i.e., the study did not achieve 50% cytotoxicity). However, the lack of a statistically significant response in the current study was not an impediment to the primary objective of developing a multiplexed, in vitro MN assay capable of MoA assessment using the ImageStream platform.

In all instances for the endpoints of both MMS and carbendazim (with the exception of the MMS endpoints G2/M, S and the carbendazim endpoints G2/M, ɣH2AX), the one-way ANOVA identified significant differences between the mean responses across dose groups (Supplementary Table ST1).

Whilst the ISMN-mb approach underestimated MN frequencies for both MMS and carbendazim compared to the manual scored cytokinesis-block micronucleus (CBMN) assay (Verma et al. 2018)—it did so consistently. The result of this is that both methods yielded near-identical BMD values upon quantitative assessment of the dose–response data (data and analysis presented in Table ST2 and Figure S1, respectively).

## Discussion

Building on previous work utilising ɣH2AX, pH3 and p53 staining as DNA damage biomarkers (Bryce et al. 2016; Dertinger et al. 2019; Khoury et al. 2016; Smart et al. 2011; Wilson et al. 2021) and MN analysis using ImageStream (Rodrigues 2018, 2019; Rodrigues et al. 2014, 2016a, b, 2018), we developed a simple and robust protocol enabling a multiplexed, imaging cytometry-based MN assay using un-lysed human TK6 cells. In combination, the Cytek® Amnis® Imaging flow cytometry platform and IDEAS® 6.2 software enabled single-cell image analysis. Cell intensity and cellular morphology features along with p53, pH3 and ɣH2AX biomarker signals and cell cycle analysis were integrated to provide MoA profiles that clearly differentiated MN formation induced by MMS or carbendazim. The distinct DNA damage profiles observed with MMS or carbendazim were consistent with other assay formats (Bryce et al. 2016, 2017; Dertinger et al. 2019; Khoury et al. 2016) and showed increases in MN and p53 signal alongside biomarker features classically associated with a clastogenic MoA as expected with MMS (increase ɣH2AX; decrease pH3) or an aneugenic MoA as expected with carbendazim treatment (decrease ɣH2AX; increase pH3).

We found the IDEAS® 6.2 software to be intuitive and user friendly. Templated gating designs were initially used to control for user bias while batch analysis provided high content outputs to mine for responses relevant to the individual DNA damage biomarkers and nuclear staining used. However, several limitations were identified with this approach. For example, because of the lack of an automated cell cycle assessment feature manual placement of cell cycle gates G1, S, and G2/M-phases was required to account for cell-cycle variability. The final micronucleus mask used could be described logically as ‘Cytoplasm AND MN but NOT Nucleus’. This mask was constructed by layering three separate MN masks defined by various software functions and image features as described in the results to account for the range of nuclear and MN phenotypes, as well as MN-assay criteria defined by Fenech et al. (2003). In addition, the mask was designed to minimise the exclusion of specific cell sub-populations from the total data set. However, based on current criteria, the masking spot count feature for MN analysis only had a mean accuracy of 57% which indicated a miss rate of 44%, and these values varied according to each chemical. Whilst the accuracy and miss rates of the ISMN-mb MN mask in IDEAS® software were just 57% and 44%, respectively, due to the consistent way in which the automated approach scored the images the dose-responses provided by the ISMN-mb assay were proportional to data collected using the gold-standard manual CBMN assay. In the revision, this is demonstrated by the achieved benchmark dose (BMD) values for manually scored versus ISMN-scored outcomes being near-identical at ~ 0.6 μg/mL (ISMN) or ~ 0.7 μg/mL (manual slide scoring) (analysis presented, Supplementary Figure S1). Despite these comparable BMD values, however, the low miss- and accuracy rates are not ideal, and demonstrates Boolean logic masking combinations in the IDEAS® system lack the finesse or sensitivity to account for all potential MN phenotypes and this is consistent with the observations of others (Rodrigues et al. 2018, 2021). Whereas an argument could be made to increase the complexity of the IDEAS® masking approach to improve these percentages, the sheer number of permutations of the mask structure this would require, without sacrificing population inclusion, is not practical. As such, in the future we plan to analyse the collected images using an advancement of the deep learning scoring methodology described by Wills et al. (2021) which will provide object masks instead of just image classifications and has already been shown to be capable of providing > 90% accuracy of classifying micronucleus events in binucleated cells.

A stepwise approach (comparing stained and unstained samples alongside fluorescent histogram figures) was used to define a gating strategy and thereby remove the subjectivity which is often associated with flow cytometry gating and cell population selection. The assessment of fluorescence intensity signals for each of the ɣH2AX, p53 and pH3 biomarkers, with defined gating cut-off values, were used to provide a measure of the biomarker response. This unbiased approach permitted dose dependant assessments of biologically relevant fold changes in biomarker responses whilst simultaneously excluding potential skewing of results as a result of autofluorescence, camera background signals, or off target antibody binding. Cross reference of the gating outputs with cell images associated with each of the scatter points provided confidence that the final gating parameters selected were robust and therefore appropriate for signal metrics to be extracted.

The major advantage of this assay is the simultaneous detection of DNA damage MoA information whilst preserving the morphological localisation of the high content data (as would be the case with classical microscopy) combined with the high throughput analysis of flow cytometry. This platform reduces the subjectivity of traditional flow cytometry gating, due to the individual assessment of physical cellular images and additional features offered within the IDEAS® software. Moreover, the relative position of histone foci and MN are maintained within the boundary of the cell membrane because the assay does not require cell lysis. This approach ensures that each MN and cellular signal is associated with its own cell of origin and, therefore, will improve assay precision. Looking forward, the morphological data that is associated with each image could be mined automatically for additional spatial information to aid MoA categorisation and differential risk assessment. For instance, further characterising aneugens (Elhajouji et al. 2011) or thresholds based on point of departure dose–response characterisation (Wills et al. 2017). The ability to analyse thousands of cells across multiple samples in mere seconds should provide the quantitative data required for such applications, which together with Artificial Intelligence (AI) imaging classifiers could contribute to the evolving landscape of next-generation genetic toxicology testing (Dearfield et al. 2017; Sasaki et al. 2020; Zeiger et al. 2015).

The user-friendly template system described in the current study provided a robust system for fast and efficient data extraction requiring minimal user input enabling unbiased assessments of chemical MoA. The analysis of additional chemicals will be reported in due course, and more work is ongoing to develop the data analytics. This future work includes signal normalisation and the use of artificial intelligence approaches to improve the accuracy of the image analysis building on recently-described advances (Rodrigues et al. 2021; Wills et al. 2021).

## Materials and methods

### Test article formulation

Master stock solutions for each chemical were made fresh on the day of the experiment in DMSO.

Methyl methanesulphonate (MMS), CAS no. 66-27-3, carbendazim, each supplied from Sigma-Aldrich. The working concentrations for MMS (0.00, 0.31, 0.63, 1.25, 2.50, 5.00 μg/mL) and carbendazim (0.00, 0.40, 0.60, 0.80, 1.20 and 1.60 μg/mL) were selected based on the data produced by Verma et al. (2017). The vehicle control was dimethylsulphoxide (DMSO) (CAS no. 67-68-5).

### Cell culture and growth media

Human, p53 competent, lymphoblastoid TK6 cells (Cat. No. 95111735, alternate collection no. ATCC CRL 8015) were used in this study and obtained from European Collection of Cell Cultures (ECACC) Salisbury (Branda et al. 2001). RPMI 1640 (Gibco) culture media supplemented with 1% penicillin streptomycin (pen strep) and 10% heat inactivated horse serum (Gibco) was used for TK6 cell culture. Cells were incubated at 37 °C in a humidified atmosphere of 5% (v/v) CO_2_. TK6 cells doubled every 16–18 h and once cells reached confluence sub-cultures were established. Each subculture did not exceed a confluence value of 1 × 10^6^ cells/mL as per ECACC/ATCC recommendations.

### Treatment of cell cultures

2 × 10^5^ TK6 cells/mL were placed in a series of sterile vented tissue culture flasks and treated with either MMS or Carbendazim for a 1.5 cell cycle period with no recovery. Dose volume to cell culture did not exceed 1% i.e., 100 μL of dose was added to 9.9 mL of cell suspension. Any precipitation or colour change was noted upon chemical addition to cell culture flask. All incubation steps occurred at 37 °C, 5% (v/v) CO_2_ ± 0.5% in air. Each replicate when performed on the same day were generated from cells of a different passage. After the treatment period, cell counts were taken for each culture using a Beckman coulter counter. Cell cultures were then transferred to 15 mL centrifuge tubes and were centrifuged at 200*g* for 8 min, supernatant was discarded, and the pellet re-suspended in 5 mL pre-warmed RPMI HIHS culture media. Subsequently, the RPMI media was removed via centrifugation at 200*g*, the pellet was re-suspended and wash step repeated with 5 mL Phosphate-Buffered Saline (PBS). The highest concentration tested was one that allowed the maximum exposure up to 2000 μg/mL or 10 mM for freely soluble test articles, or the limit of solubility or toxicity, whichever is lower (OECD 2023). Where toxicity was a limiting factor, the maximum treatment concentration selected for analysis was that with relative cell growth at 30%.

### Cell counts and cytotoxicity

Cells were counted using a Beckman Coulter Counter and Relative Cell Growth (RCG) was used to estimate cytotoxicity in treated samples. RCG was calculated as follows:

$$\text{RCG}= \frac{\text{No}.\,\text{cells}/\text{mL\,in\,treated\,culture}}{\text{No}.\,\text{cells}/\text{mL\,in\,vehicle\,control }} \times 100$$

### Cell fixation and staining

Following cell treatment period wash steps, the cell pellet was resuspended in residual PBS and BD FACS Lyse was used to fix and permeabilise the cells. BD FACSLyse was diluted in a 1:10 ratio with distilled water (dH_2_O) (1 mL FACS Lyse: 9 mL dH_2_O). Avoiding further agitation of samples, 2 mL of FACS Lyse solution was added to each sample. Samples were incubated at room temperature for 12 min. Following incubation samples were centrifuged at 200*g* for 5 min. The supernatant was discarded and the cell pellet gently resuspended in the remaining solution, 5 mL PBS was added and samples returned to centrifuge for further 5 min, this wash step was repeated twice. Samples at this stage may be placed in the fridge and stained at a later date or stained immediately. Samples were stained with 300 μL of antibody (AB) master mix for a minimum of 60 min under agitation at room temperature. The master mix consisted of BV421 anti-ɣH2AX AB (Cat. No. 564720, BD Biosciences), AF488 anti-pH3 AB (Cat. No. 641003, BioLegend) and PE anti-p53 AB (Cat.No. 645805, BioLegend) in the ratio 3 μL of pH 3: 5 μL of ɣH2AX: 6 μL of p53: 286 μL of PBS. DRAQ5™ DNA stain (Cat. No. 564902, BD Biosciences) was used to label nuclei and MN. 2 μL DRAQ5™:98 μL PBS was mixed, 100 μL of the 1:49 ratio stain solution added to each 300 μL cell sample antibody solution making final staining ratio of 1:199. Samples were counterstained with DRAQ5™ for a minimum of 20 min. After the staining period ended samples were centrifuged and washed in 5 mL PBS.

### ImageStream X Mark II® data acquisition

Samples were analysed on a Cytek® Amnis® ImageStream®X Mk IIimaging flow cytometer using Cytek® Amnis® INSPIRE® software version 6.2 (Merck Millipore, Nottingham UK). Prior to experimental analysis, following the manufacturer’s instructions for appropriate ImageStream X Mark II® set up and quality control procedures, all system calibrations performed and passed using ImageStream X Mark II® SpeedBead calibration reagents (Cat. No. 400041).

100 µL aliquots of cell suspension at a concentration of ~ 7 × 10^5^ and no less than ~ 4 × 10^5^ cells/mL were prepared in 1.5 mL Eppendorfs. Replicate 1 and 2 for both MMS and Carbendazim treated cell samples were analysed by placing the Eppendorfs on the sample port and a minimum of 50µl sample loaded. For replicate 3 of both chemicals, cell samples were transferred to a 96-well plate. Prior to data collection laser intensities were balanced to limit saturation events and fluorescence channel overspill. For data acquisition an INSPIRE® template was set up for in focus and single cellular event gating, using Aspect Ratio and Root Mean Square (RMS) features, ensuring collected cells were sufficiently circular and in focus. Once established, data were acquired at a low velocity 66.0 (mm/s), resulting in 15,000–30,000 single cellular events being collected in approximately 45 s at a magnification of × 40, and subsequently automatically saved for each experimental replicate. Acquisition of images for ɣH2AX pH3, p53 and DRAQ5™ assessment occurred in: Channel 1 and 9, bright field; channel 2, 3, 7 and 11 fluorescence; Channel 6 side scatter. Whilst these channels were of main interest data was acquired for all 12 channels. All samples were collected with no compensation applied.

Compensation sample files were acquired at the same time as sample analysis using the INSPIRE® acquisition compensation wizard or manually using Compensation beads (Cat.No.01-2222-41) from Thermofisher Scientific. Acquisition of compensation samples was performed without the presence of brightfield or side scatter. 488, 405 and 642 nm lasers were utilised at the same intensity values used during the experimental setup. Acquired files formed compensation matrices in Cytek® Amnis® IDEAS® 6.2 software.

### IDEAS® 6.2 data analysis

Once data were collected for all samples, the acquired raw image files (.RIF) data files were analysed using IDEAS® v6.2 software (Merck Millipore, Nottingham, UK). Compensation matrices and template analyses were applied enabling generation of subsequent.CIF (compensated image file) and .DAF (data analysis file) files.

Use of a standardised analysis template allowed for batch processing of data files and extraction of the following metrics: Cell cycle, MN, ɣH2AX, pH3 and p53. Due to the slight shifting of the DNA cell cycle histogram the position of the cell cycle gates (G1, S, G2/M) were adjusted accordingly per sample. The finalised template used the following generated masks: Brightfield 1A, Brightfield 1B, Cytoplasm, Nuclear Mask 1, Nuclear Mask 2, Nuclear Mask 3, gH2AX mask, Nuclear gH2AX mask, pH3 Mask, Nuclear pH3 Mask, P53 Mask, P53 Cytoplasmic Mask, P53 Nuclear mask and Complete Final MN Mask (CFM). The functions in various combinations used to generate these masks were morphology, adaptive erode, threshold, range, dilate, intensity, spot, watershed and level set. Features in the IDEAS® system are mathematical expressions that assess, within the image, quantitative and spatial information. These allow for the generation of histograms and scatter graphs in the analysis area for cell population responses to be assessed. The features used were: Aspect ratio, Area, contrast, Gradient Root Mean Square (RMS), Intensity, Similarity, and spot. The mask development and features used were based on the recommendations within the IDEAS® 6.2 user manual 2015, the publication Imaging Flow Cytometry: Methods and Protocols 2016 and literature (Filby et al. 2016; Patterson et al. 2015; Rodrigues et al. 2018; Verma et al. 2018). Details of the various masks, features and functions used can be found in the example template file provided for download at the BioStudies database under accession number S-BSST1351. For additional clarity, Supplementary Table 3 (ST3) demonstrates the image function combinations used to generate the three masks for the CFM.

### Cell populations used for analysis

Cells that are circular, single and in focus were determined by meeting the criteria of having an aspect ratio of >  ~ 0.6, brightfield area of >  ~ 70 but <  ~ 450 and gradient root mean square value > 50, respectively (Filby et al. 2016; Rodrigues et al. 2018; Verma et al. 2018). This single and in focus cell population was then assessed for cell health. The healthy cell population was determined by combining the nuclear area feature of nuclear mask 1 with the contrast feature applied to the cytoplasm mask. Healthy cells have a high nuclear area with pixels that meet the 50% threshold intensity and low cytoplasmic contrast (Filby et al. 2016, 2011; Rodrigues 2018; Rodrigues et al. 2018). A healthy cell population with DNA content showing > 1 × 10^5^ nuclear intensity was used for pH3 and p53 assessment. The cell population used for ɣH2AX, and MN assessment was also required to be negative for pH3 staining. To determine the mononucleated cell population for MN assessment nuclear mask 3 was used in combination with the spot count feature. This generated a histogram from which healthy mononucleated cells could be extracted.

### Biomarker and MN metric extraction

Gating parameters for ɣH2AX pH3 and p53 were determined based on the cell populations of unstained vehicle and stained vehicle samples compared to unstained and stained genotoxicant dosed samples on intensity histograms. Signal separation on scatter graphs and specific signal masking based on nuclear/cytoplasmic localization further refined the gating strategy. Development of the gating strategy is described in the results section. A minimum of ~ 13,000 single in focus healthy cells were assessed per dose per replicate to obtain the dose response for each biomarker.

The mononucleated healthy cell population was used to extract the MN data through applying the spot count feature to the complete final micronucleus mask. This automatically extracted the cell population containing MN. Development of the complete final micronucleus mask is described in the results section and applies the MN criteria for slide based scoring (Fenech et al. 2003). A minimum of ~ 10,000 mononucleated cells were assessed per dose, per replicate to obtain the MN dose response.

### Dose–response comparison

Covariate BMD analysis using PROAST 71.1 in the R programming environment (version 4.2.1) was performed for carbendazim collected either by the ISMN-mb approach or scored manually from slides (manual data obtained from Verma et al. 2018). The results of this analysis are presented in Figure S1.

### Micronucleus mask accuracy

To understand the effectiveness of the MN mask generated, two metrics were assessed based on the MN populations of four tool compounds at the highest analysable concentration. The first, ‘percentage accuracy’ (%Accuracy), is described as; of the cell population Identified by the MN mask as containing MN what percentage were true, this was confirmed by inspecting each saved cell image by eye. %Accuracy was calculated as follows:

$$\%\text{Accuracy}= \frac{\text{Total\,True\,MN\,identified\,by\,mask}}{\text{Total\,cells\,with\,MN\,identified\,by\,mask }} \times 100$$

The second, ‘percentage Miss Rate’ (%Miss Rate) required a defined cell population where images could be assessed both by eye and using the MN mask. This population was selected by plotting the healthy mononucleated cell population as a histogram using the Gradient RMS feature on the DNA content. A gate termed DNA Focus was drawn to the right of the central point of the in-focus nuclear content histogram. This population taken forward provided cells with clear crisp nuclei staining, giving the MN mask the best chance of identifying all MN, whilst randomly selecting cells to help minimize technician population bias and still being representative of the whole cell population. The total number of cells assessed were 1000–1500 per data file. %Miss Rate was calculated as follows:

$$\%\text{Miss\,Rate}= \frac{\text{Total\,True\,MN\,identified\,by\,mask}}{\text{Total\,True\,MN\,identified\,manually }} \times 100$$

### Data evaluation criteria

All tables and graphs were generated using Excel Microsoft. Unless otherwise stated data displayed in tables and plotted on graphs for biomarker and MN metrics are presented in raw response fold change that has been square-rooted to normalise. Cell cycle data are presented as raw response cell percentages. Fold change cut offs were also applied to graphical data in line with industry standards. It is important to note, the fold changes have been taken from the literature for expediency and specific cut off values for the ImageStream platform have not yet been defined. For pH3 this was 1.3 fold increase and 0.7 fold decrease when compared to vehicle controls (Khoury et al. 2016). For ɣH2AX and p53 fold change increase of 1.5 when compared to vehicle controls was used (Dertinger et al. 2019; Smart et al. 2011). These fold change values were square-rooted giving values of: 1.2 for ɣH2AX and p53. 1.1 for pH3 for increase in signal and 0.8 for decrease in signal. The MN response was considered positive based on a statistically significant response (*p* < 0.05) compared to that of vehicle control (Johnson et al. 2014). Where statistical significance was not achieved the MN response was considered positive based on a greater than twofold increase compared to control (Shi et al. 2010; Takeiri et al. 2019), this was then square-rooted giving a fold change value threshold of 1.4. A genotoxic response is considered positive when the mean response relative to control exceeds the fold change cut off or is statistically significant. Where both MN and biomarker response exceed fold change cut offs and/or are statistically significant indication of MoA may be inferred (Bryce et al. 2017; Dertinger et al. 2019).

### Statistical analysis

Dose–response data were tested for variance homogeneity and normality using Bartlett and Shapiro–Wilk tests, respectively. Where datasets ‘passed’ these tests (i.e., *p* > 0.05), a one-way ANOVA was run followed by pairwise testing versus control to establish response significance (*p* ≤ 0.05) carried out by Dunnett’s *T*-test method. In the event the data failed the distribution tests the non-parametric Dunn’s test was performed on the raw response data. All statistics were calculated using DRSMOOTH package in the R programming environment using methods described in Johnson et al. (2014).

### Supplementary Information

Below is the link to the electronic supplementary material.

Supplementary file1 (PPTX 79 KB)

## Data Availability

Imaging flow cytometry test file and compensation matrix, alongside the final IDEAS® template are provided for download from the BioStudies database (https://www.ebi.ac.uk/biostudies/studies/S-BSST1351) under accession number S-BSST1351.
