# Supplementary material for: A multi-biomarker micronucleus assay using imaging flow cytometry
Source: Arch Toxicol. 2024 Jul 12;98(9):3137–53. doi: 10.1007/s00204-024-03801-7 (PMC11324684; doi:10.1007/s00204-024-03801-7)
Supplement: Supplementary file 1 — Supplementary file1 (PPTX 79 KB) [file 204_2024_3801_MOESM1_ESM.pptx]

## Slide 1
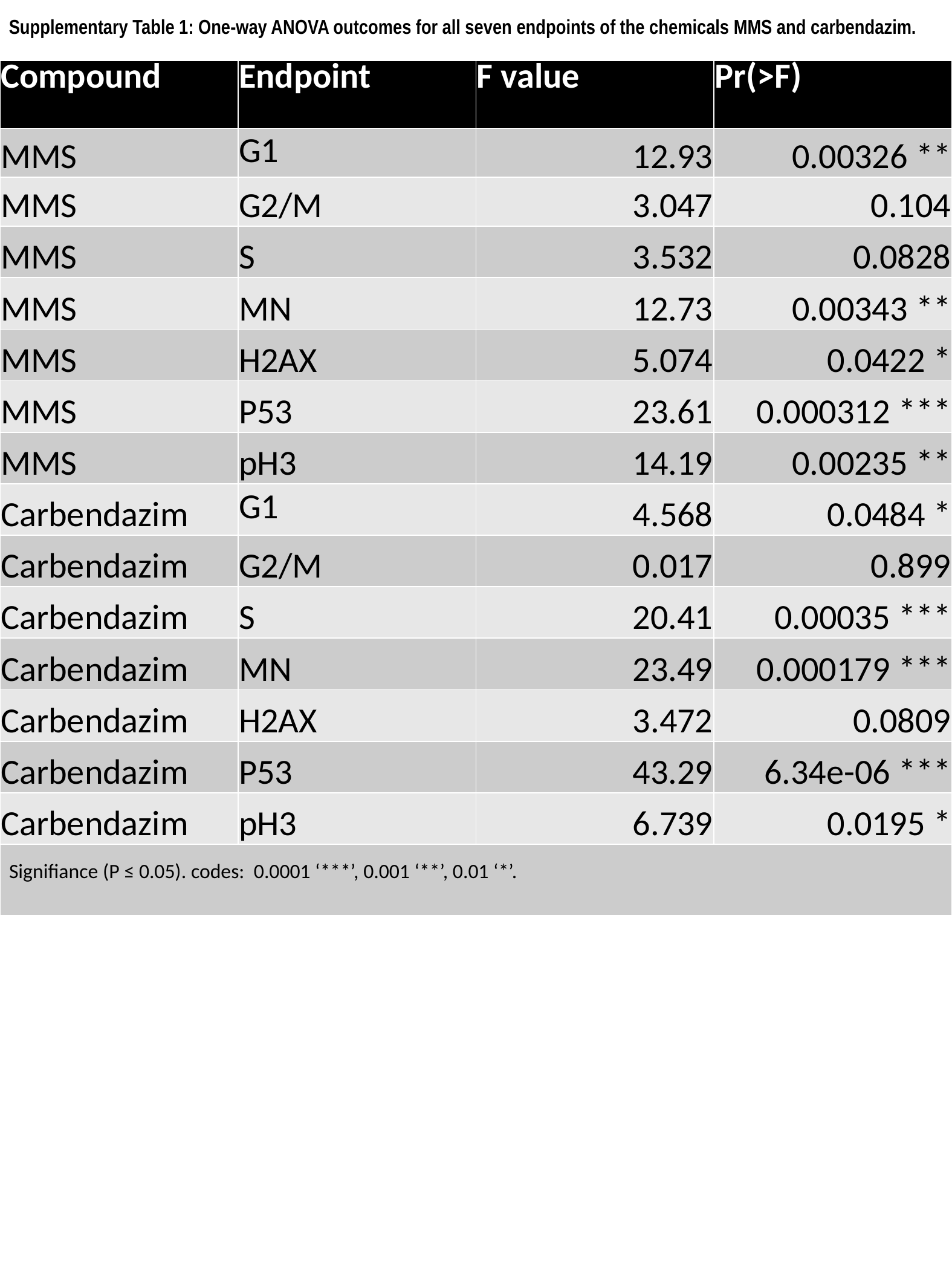

Supplementary Table 1: One-way ANOVA outcomes for all seven endpoints of the chemicals MMS and carbendazim.
| Compound | Endpoint | F value | Pr(>F) |
| --- | --- | --- | --- |
| MMS | G1 | 12.93 | 0.00326 \*\* |
| MMS | G2/M | 3.047 | 0.104 |
| MMS | S | 3.532 | 0.0828 |
| MMS | MN | 12.73 | 0.00343 \*\* |
| MMS | H2AX | 5.074 | 0.0422 \* |
| MMS | P53 | 23.61 | 0.000312 \*\*\* |
| MMS | pH3 | 14.19 | 0.00235 \*\* |
| Carbendazim | G1 | 4.568 | 0.0484 \* |
| Carbendazim | G2/M | 0.017 | 0.899 |
| Carbendazim | S | 20.41 | 0.00035 \*\*\* |
| Carbendazim | MN | 23.49 | 0.000179 \*\*\* |
| Carbendazim | H2AX | 3.472 | 0.0809 |
| Carbendazim | P53 | 43.29 | 6.34e-06 \*\*\* |
| Carbendazim | pH3 | 6.739 | 0.0195 \* |
| Signifiance (P ≤ 0.05). codes: 0.0001 ‘\*\*\*’, 0.001 ‘\*\*’, 0.01 ‘\*’. | | | |

## Slide 2
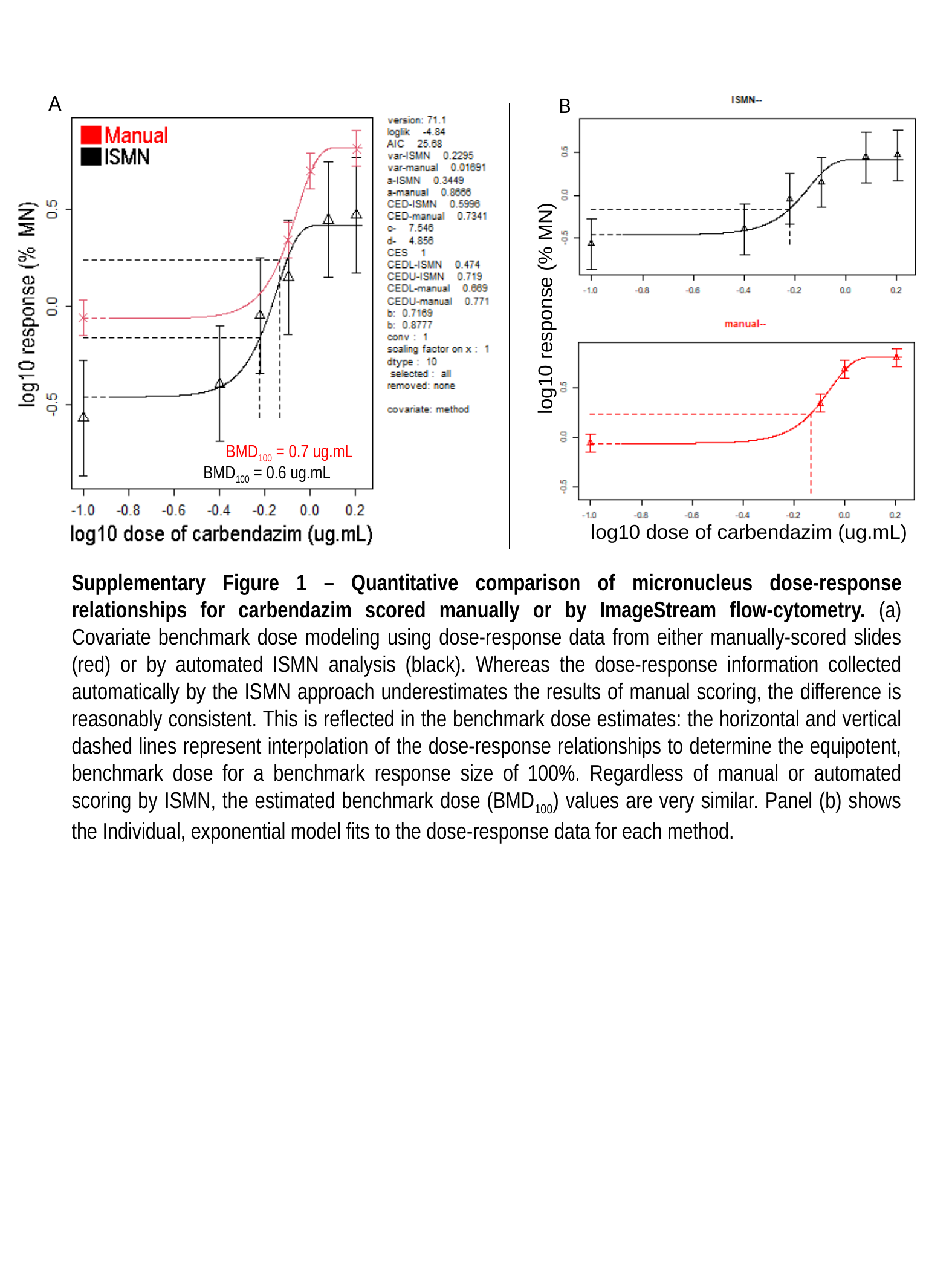

A
B
log10 response (% MN)
log10 dose of carbendazim (ug.mL)
BMD100 = 0.7 ug.mL
BMD100 = 0.6 ug.mL
Supplementary Figure 1 – Quantitative comparison of micronucleus dose-response relationships for carbendazim scored manually or by ImageStream flow-cytometry. (a) Covariate benchmark dose modeling using dose-response data from either manually-scored slides (red) or by automated ISMN analysis (black). Whereas the dose-response information collected automatically by the ISMN approach underestimates the results of manual scoring, the difference is reasonably consistent. This is reflected in the benchmark dose estimates: the horizontal and vertical dashed lines represent interpolation of the dose-response relationships to determine the equipotent, benchmark dose for a benchmark response size of 100%. Regardless of manual or automated scoring by ISMN, the estimated benchmark dose (BMD100) values are very similar. Panel (b) shows the Individual, exponential model fits to the dose-response data for each method.

## Slide 3
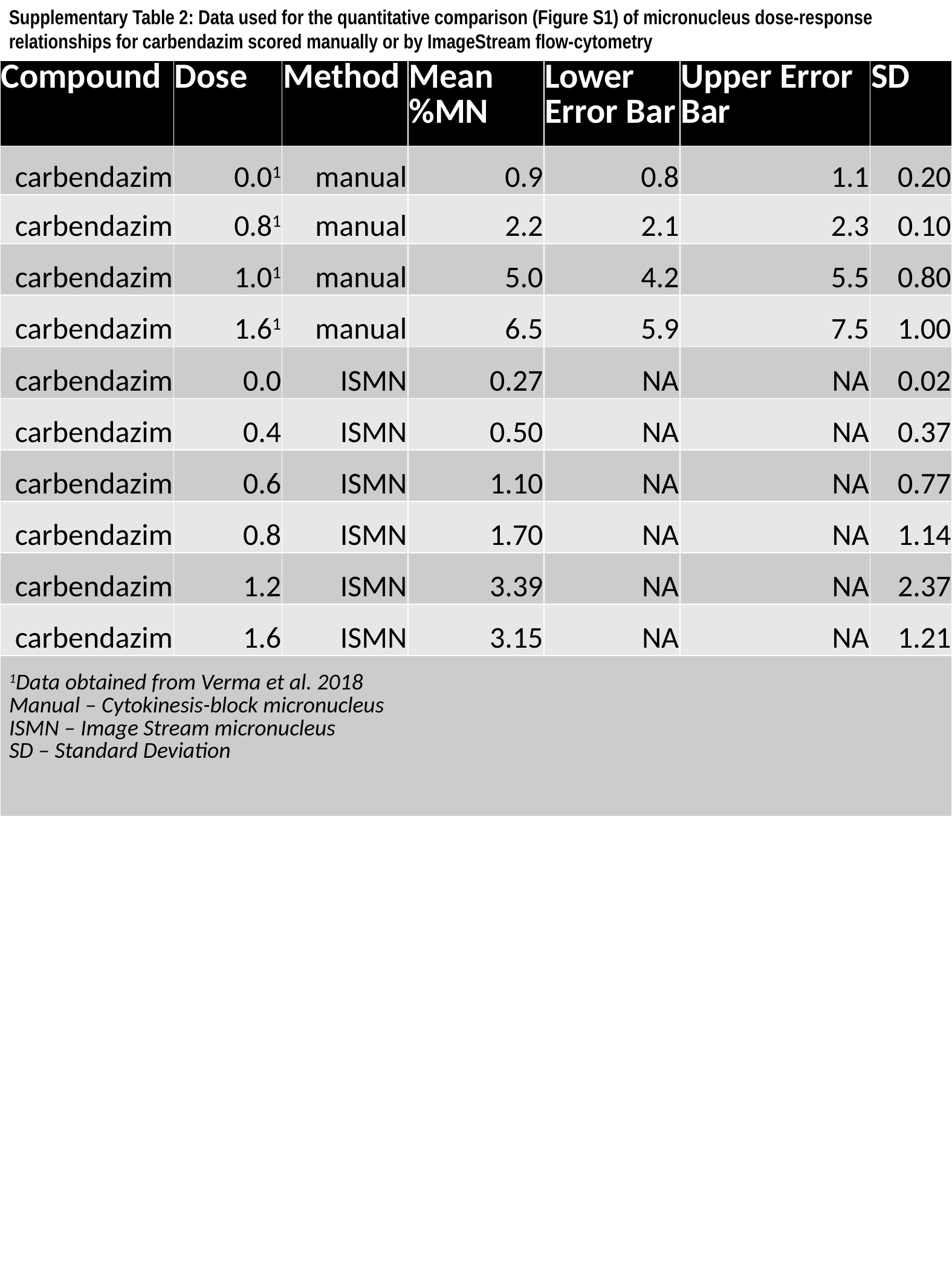

Supplementary Table 2: Data used for the quantitative comparison (Figure S1) of micronucleus dose-response relationships for carbendazim scored manually or by ImageStream flow-cytometry
| Compound | Dose | Method | Mean %MN | Lower Error Bar | Upper Error Bar | SD |
| --- | --- | --- | --- | --- | --- | --- |
| carbendazim | 0.01 | manual | 0.9 | 0.8 | 1.1 | 0.20 |
| carbendazim | 0.81 | manual | 2.2 | 2.1 | 2.3 | 0.10 |
| carbendazim | 1.01 | manual | 5.0 | 4.2 | 5.5 | 0.80 |
| carbendazim | 1.61 | manual | 6.5 | 5.9 | 7.5 | 1.00 |
| carbendazim | 0.0 | ISMN | 0.27 | NA | NA | 0.02 |
| carbendazim | 0.4 | ISMN | 0.50 | NA | NA | 0.37 |
| carbendazim | 0.6 | ISMN | 1.10 | NA | NA | 0.77 |
| carbendazim | 0.8 | ISMN | 1.70 | NA | NA | 1.14 |
| carbendazim | 1.2 | ISMN | 3.39 | NA | NA | 2.37 |
| carbendazim | 1.6 | ISMN | 3.15 | NA | NA | 1.21 |
| 1Data obtained from Verma et al. 2018 Manual – Cytokinesis-block micronucleus ISMN – Image Stream micronucleus SD – Standard Deviation | | | | | | |

## Slide 4
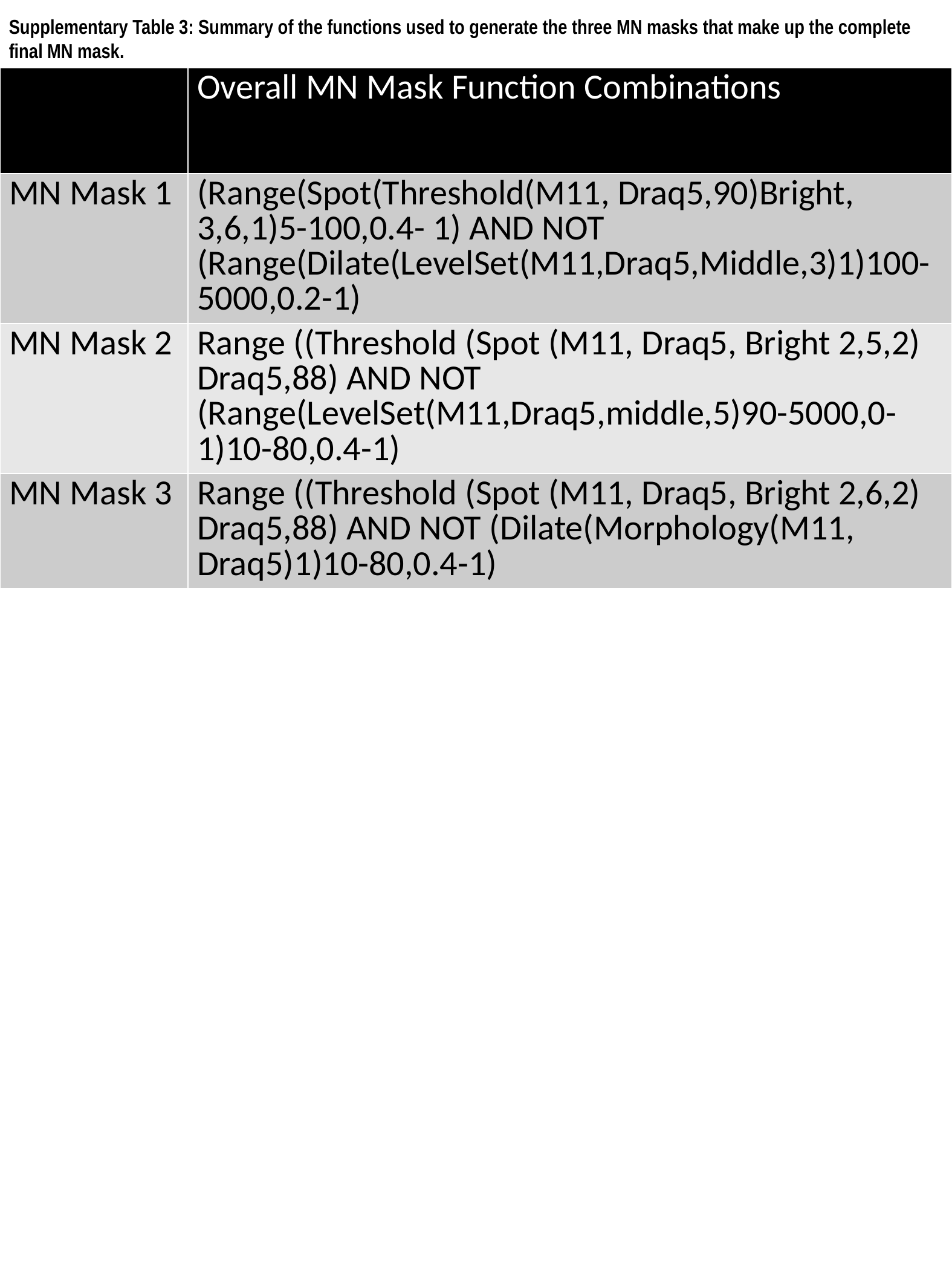

Supplementary Table 3: Summary of the functions used to generate the three MN masks that make up the complete final MN mask.
| | Overall MN Mask Function Combinations |
| --- | --- |
| MN Mask 1 | (Range(Spot(Threshold(M11, Draq5,90)Bright, 3,6,1)5-100,0.4- 1) AND NOT (Range(Dilate(LevelSet(M11,Draq5,Middle,3)1)100- 5000,0.2-1) |
| MN Mask 2 | Range ((Threshold (Spot (M11, Draq5, Bright 2,5,2) Draq5,88) AND NOT (Range(LevelSet(M11,Draq5,middle,5)90-5000,0-1)10-80,0.4-1) |
| MN Mask 3 | Range ((Threshold (Spot (M11, Draq5, Bright 2,6,2) Draq5,88) AND NOT (Dilate(Morphology(M11, Draq5)1)10-80,0.4-1) |
